# Supplementary material for: Molecular mimicry in multisystem inflammatory syndrome in children
Source: Nature. 2024 Aug 7;632(8025):622–9. doi: 10.1038/s41586-024-07722-4 (PMC11324515; doi:10.1038/s41586-024-07722-4)
Supplement: Supplementary file 1 — The complete set of primers used for the single-cell T cell receptor (TCR) sequencing by nested multiplex polymerase chain reaction (PCR). [file 41586_2024_7722_MOESM1_ESM.pdf]

nature portfolio

https://doi.org/10.1038/s41586-024-07722-4

Supplementary information

Molecular mimicry in multisystem inflammatory syndrome in children

In the format provided by the authors and unedited

Supplementary Table 1: Primers for single-cell TCR sequencing by nested multiplex PCR

Primer Type	Primer Name	Primer Sequence[1]
**Forward (External)**		
	hTRAV1-ext	AACTGCACGTACCAGACATC
	hTRAV2-ext	GATGTGCACCAAGACTCC
	hTRAV3-ext	AAGATCAGGTCAACGTTGC
	hTRAV4-ext	CTCCATGGACTCATATGAAGG
	hTRAV5-ext	CTTTTCCTGAGTGTCCGAG
	hTRAV6-ext	CACCCTGACCTGCAACTATAC
	hTRAV7-ext	GCAAAATACAGGGATGGG
	hTRAV8-1-ext	CTCACTGGAGTTGGGATG
	hTRAV8-3-ext	CACTGTCTCTGAAGGAGCC
	hTRAV8-2,4-ext	GCCACCCTGGTTAAAGG
	hTRAV8-6-ext	GAGCTGAGGTGCAACTACTC
	hTRAV8-7-ext2	CTAACAGAGGCCACCCAG
	hTRAV9-1_2-ext	TGGTATGTCCAATATCCTGG
	hTRAV10-ext	CAAGTGGAGCAGAGTCCTC
	hTRAV12-1_3-ext	CARTGTTCCAGAGGGAGC
	hTRAV13-1-ext	CATCCTTCAACCCTGAGTG
	hTRAV13-2-ext	CAGCGCCTCAGACTACTTC
	hTRAV14-ext	AAGATAACTCAAACCCAACCAG
	hTRAV16-ext	AGTGGAGCTGAAGTGCAAC
	hTRAV17-ext	GGAGAAGAGGATCCTCAGG
	hTRAV18-ext3	TCCAGTATCTAAACAAAGAGCC
	hTRAV19-ext	AGGTAACTCAAGCGCAGAC
	hTRAV20-ext	CACAGTCAGCGGTTTAAGAG
	hTRAV21-ext	TTCCTGCAGCTCTGAGTG
	hTRAV22-ext	GTCCTCCAGACCTGATTCTC
	hTRAV23-ext	TGCTTATGAGAACACTGCG
	hTRAV24-ext	CTCAGTCACTGCATGTTCAG
	hTRAV25-ext	GGACTTCACCACGTACTGC
	hTRAV26-1-ext	GCAAACCTGCCTTGTAATC
	hTRAV26-2-ext	AGCCAAATTCAATGGAGAG
	hTRAV27-ext	TCAGTTTCTAAGCATCCAAGAG
	hTRAV29-ext	GCAAGTTAAGCAAAATTCACC

hTRAV30-ext	CAACAACCAGTGCAGAGTC
hTRAV34-ext	AGAACTGGAGCAGAGTCCTC
hTRAV35-ext	GGTCAACAGCTGAATCAGAG
hTRAV36-ext	GAAGACAAGGTGGTACAAAGC
hTRAV38-ext	GCACATATGACACCAGTGAG
hTRAV39-ext	CTGTTCCTGAGCATGCAG
hTRAV40-ext	GCATCTGTGACTATGAACTGC
hTRAV41-ext	AATGAAGTGGAGCAGAGTCC
hTRBV2-ext	TCGATGATCAATTCTCAGTTG
hTRBV3-ext	CAAAATACCTGGTCACACAG
hTRBV4-ext	TCGCTTCTCACCTGAATG
hTRBV5-1_4-ext	GATTCTCAGGKCKCCAGTTC
hTRBV5-5_8-ext	GTACCAACAGGYCCTGGGT
hTRBV6-1_3,5_9-ext	ACTCAGACCCCAAAATTCC
hTRBV6-4-ext	ACTGGCAAAGGAGAAGTCC
hTRBV7-1_3-ext	TRTGATCCAATTTCAGGTCA
hTRBV7-4_9-ext new	CGSWTCTYTGCAGARAGGC
hTRBV9-ext	GATCACAGCAACTGGACAG
hTRBV10-1-ext	CAGAGCCCAAGACACAAG
hTRBV10-2-ext	ACCTTGATGTGTCACCAGAC
hTRBV10-3-ext	CAGAGCCCAAGACACAAG
hTRBV11-ext	CGATTTTCTGCAGAGACGC
hTRBV12-ext	ARGTGACAGARATGGGACAA
hTRBV13-ext	AGCGATAAAGGAAGCATCC
hTRBV14-ext	CCAACAATCGATTCTTAGCTG
hTRBV15-ext	AGTGACCCTGAGTTGTTCTC
hTRBV16-ext	GTCTTTGATGAAACAGGTATGC
hTRBV17-ext	CAGACCCCCAGACACAAG
hTRBV18-ext	CATAGATGAGTCAGGAATGCC
hTRBV19-ext	AGTTGTGAACAGAATTTGAACC
hTRBV20-ext	AAGTTTCTCATCAACCATGC
hTRBV23-ext	GCGATTCTCATCTCAATGC
hTRBV24-ext	CCTACGGTTGATCTATTACTCC
hTRBV25-ext	ACTACACCTCATCCACTATTCC
hTRBV27,28-ext	TGGTATCGACAAGACCCAG
hTRBV29-ext	TTCTGGTACCGTCAGCAAC
hTRBV30-ext	TCCAGCTGCTCTTCTACTCC

**Reverse (External)**		
	hTRAC-ext	GACCAGCTTGGACATCACAG
	hTRBC-ext	TAGAACTGGACTTGACAGCG
**Forward (Internal)**		
	hTRAV1-int	GCACCCACATTTCTKTCTTAC
	hTRAV2-int	CACTCTGTGTCCAATGCTTAC
	hTRAV3-int	ATGCACCTATTCAGTCTCTGG
	hTRAV4-int	ATTATATCACGTGGTACCAACAG
	hTRAV5-int	TACACAGACAGCTCCTCCAC
	hTRAV6-int	TGGTACCGACAAGATCCAG
	hTRAV7-int	TATGAGAAGCAGAAAGGAAGAC
	hTRAV8-1-int	GTCAACACCTTCAGCTTCTC
	hTRAV8-2,8-4-int	TTTGAGGCTGAATTTAAGAGG
	hTRAV8-3-int	AGAGTGAAACCTCCTTCCAC
	hTRAV8-6-int	AACCAAGGACTCCAGCTTC
	hTRAV8-7-int	ATCAGAGGTTTTGAGGCTG
	hTRAV9-1,9-2-int	GAAACCACTTCTTTCCACTTG
	hTRAV10-int	GAAAGAACTGCACTCTTCAATG
	hTRAV12-1,12-2,12-3-int	AAGATGGAAGGTTTACAGCAC
	hTRAV13-1-int	TCAGACAGTGCCTCAAACTAC
	hTRAV13-2-int	CAGTGAAACATCTCTCTCTGC
	hTRAV14-int	AGGCTGTGACTCTGGACTG
	hTRAV16-int	GTCCAGTACTCCAGACAACG
	hTRAV17-int	CCACCATGAACTGCAGTTAC
	hTRAV18-int	TGACAGTTCCTTCCACCTG
	hTRAV19-int	TGTGACCTTGGACTGTGTG
	hTRAV20-int	TCTGGTATAGGCAAGATCCTG
	hTRAV21-int	AACTTGGTTCTCAACTGCAG
	hTRAV22-int	CTGACTCTGTGAACAATTTGC
	hTRAV23-int	TGCATTATTGATAGCCATACG
	hTRAV24-int	TGCCTTACACTGGTACAGATG
	hTRAV25-int	TATAAGCAAAGGCCTGGTG
	hTRAV26-1-int	CGACAGATTCACTCCCAG
	hTRAV26-2-int	TTCACTTGCCTTGTAACCAC
	hTRAV27-int	CTCACTGTGTACTGCAACTCC

hTRAV29-int	CTGCTGAAGGTCCTACATTC
hTRAV30-int	AGAAGCATGGTGAAGCAC
hTRAV34-int	ATCTCACCATAAACTGCACG
hTRAV35-int	ACCTGGCTATGGTACAAGC
hTRAV36-int	ATCTCTGGTTGTCCACGAG
hTRAV38-int	CAGCAGGCAGATGATTCTC
hTRAV39-int	TCAACCACTTCAGACAGACTG
hTRAV40-int	GGAGGCGGAAATATTAAAGAC
hTRAV41-int	TTGTTTATGCTGAGCTCAGG
hTRBV2-int	TTCACTCTGAAGATCCGGTC
hTRBV3-int	AATCTTCACATCAATTCCCTG
hTRBV4-int	CCTGCAGCCAGAAGACTC
hTRBV5-1,5-2,5-3,5-4-int	CTTGGAGCTGGRSGACTC
hTRBV5-5,5-6,5-7,5-8-int	TCTGAGCTGAATGTGAACG
hTRBV6-1,6-2,6-3,6-5,6-6,6-7,6-8,6-9-int	GTGTRCCCAGGATATGAACC
hTRBV6-4-int	TGGTTATAGTGTCTCCAGAGC
hTRBV7-1,7-2,7-3-int	TCYACTCTGAMGWTCCAGCG
hTRBV7-4,7-5,7-6,7-7,7-8,7-9-int	TGRMGATYCAGCGCACA
hTRBV9-int	GTACCAACAGAGCCTGGAC
hTRBV10-1-int	TGGTATCGACAAGACCTGG
hTRBV10-2-int	TGGTATCGACAAGACCTGG
hTRBV10-3-int	GGAACACCAGTGACTCTGAG
hTRBV11-int	GACTCCACTCTCAAGATCCA
hTRBV12-int	CYACTCTGARGATCCAGCC
hTRBV13-int	CATTCTGAACTGAACATGAGC
hTRBV14-int	ATTCTACTCTGAAGGTGCAGC
hTRBV15-int	ATAACTTCCAATCCAGGAGG
hTRBV16-int	CTGTAGCCTTGAGATCCAGG
hTRBV17-int	TGTTCACTGGTACCGACAG
hTRBV18-int	CGATTTTCTGCTGAATTTCC
hTRBV19-int	TTCCTCTCACTGTGACATCG
hTRBV20-int	ACTCTGACAGTGACCAGTGC
hTRBV23-int	GCAATCCTGTCCTCAGAAC
hTRBV24-int	GATGGATACAGTGTCTCTCGA
hTRBV25-int	CAGAGAAGGGAGATCTTTCC

	hTRBV27,28-int	TTCYCCCTGATYCTGGAGTC
	hTRBV29-int	TCTGACTGTGAGCAACATGAG
	hTRBV30-int	AGAATCTCTCAGCCTCCAGAC
**Reverse (internal) index[2]**		
	Hum_Acj TRAC INDEX	CGACTCAAGTGTGTGG**NNNNNN** GGGTCAGGGTTCTGGATAT
	Hum_Bcj TRBC INDEX	CGACTCAGATTGGTAC**NNNNNN** ACACSTTKTTCAGGTCCTC

[1] 5’-3’ sense primer targeting human TRAV (TCR variable α) and TRBV (TCR variable β); 3’-5’ anti-sense primers targeting human TRAC (TCR constant α) and human TRBC (TCR constant β)

[2] Well-specific index barcode (bold); C-segment-specific sequence (underlined)
