# Supplementary material for: Molecular mimicry in multisystem inflammatory syndrome in children
Source: Nature. 2024 Aug 7;632(8025):622–9. doi: 10.1038/s41586-024-07722-4 (PMC11324515; doi:10.1038/s41586-024-07722-4)
Supplement: Supplementary file 2 — Reporting Summary [file 41586_2024_7722_MOESM2_ESM.pdf]

nature portfolio

Corresponding author(s): Joseph L. DeRisi and Mark S. Anderson

Last updated by author(s): 05/27/2024

Reporting Summary

Nature Portfolio wishes to improve the reproducibility of the work that we publish. This form provides structure for consistency and transparency in reporting. For further information on Nature Portfolio policies, see our Editorial Policies and the Editorial Policy Checklist.

Statistics

For all statistical analyses, confirm that the following items are present in the figure legend, table legend, main text, or Methods section.

n/a	Confirmed	
☐	☒	The exact sample size (n) for each experimental group/condition, given as a discrete number and unit of measurement
☐	☒	A statement on whether measurements were taken from distinct samples or whether the same sample was measured repeatedly
☐	☒	The statistical test(s) used AND whether they are one- or two-sided<br>*Only common tests should be described solely by name; describe more complex techniques in the Methods section.*
☒	☐	A description of all covariates tested
☐	☒	A description of any assumptions or corrections, such as tests of normality and adjustment for multiple comparisons
☐	☒	A full description of the statistical parameters including central tendency (e.g. means) or other basic estimates (e.g. regression coefficient) AND variation (e.g. standard deviation) or associated estimates of uncertainty (e.g. confidence intervals)
☐	☒	For null hypothesis testing, the test statistic (e.g. F, t, r) with confidence intervals, effect sizes, degrees of freedom and P value noted<br>*Give P values as exact values whenever suitable.*
☒	☐	For Bayesian analysis, information on the choice of priors and Markov chain Monte Carlo settings
☒	☐	For hierarchical and complex designs, identification of the appropriate level for tests and full reporting of outcomes
☒	☐	Estimates of effect sizes (e.g. Cohen's d, Pearson's r), indicating how they were calculated

Our web collection on statistics for biologists contains articles on many of the points above.

Software and code

Policy information about availability of computer code

Data collection	RAPSearch2.0 was used to align all Illumina generated PhIP-Seq Fastq files. Flow cytometry data were collected using FACSDiva v8.01 Software (Becton Dickinson) or SpectroFlow v2.2 software (Cytek).
Data analysis	Python 3 and R v3.6.0 were used for data analysis. For the machine learning logistic regression classifier, the Scikit-learn Python package was utilized, and is referenced in the "PhIP-Seq Analysis" section of the methods, and a previous publication using this analysis is cited. For TCR sequencing and repertoire analysis, the TCRdist algorithm implementation from the CoNGA v0.1.2 python package was used. Stitchr vl.0.0 was used to reconstruct TCR sequences. Further analysis was performed using R, with merging and subsetting of data performed using the dplyr packages. TCR similarity networks were built using stringdist v0.9.12 and igraph v2.0.3 R packages and visualized using gephi v0.9.7 software. Visualizations in R was performed using ggplot2 v3.4.0 and ggpubr v0.5.0. Cell population gating and fluorescence analysis was performed using FlowJo version 10.7.2 software (BD Biosciences). Any figures created with BioRender.com were exported under a paid subscription with an associated publication license.

For manuscripts utilizing custom algorithms or software that are central to the research but not yet described in published literature, software must be made available to editors and reviewers. We strongly encourage code deposition in a community repository (e.g. GitHub). See the Nature Portfolio guidelines for submitting code & software for further information.

Data

Policy information about availability of data

All manuscripts must include a data availability statement. This statement should provide the following information, where applicable:

- Accession codes, unique identifiers, or web links for publicly available datasets
- A description of any restrictions on data availability
- For clinical datasets or third party data, please ensure that the statement adheres to our policy

The published article includes all datasets generated or analyzed as a part of this study. Individual source data are provided with associated figures (where appropriate) per the data sharing agreement stipulated under the Ruth L. Kirschstein National Research Service Award Individual Postdoctoral Fellowship (award no. F32AI157296; R.C.M.). Raw flow cytometry source files can be made available upon reasonable request. Source data are provided with this paper. All PhIP-Seq data are publicly available via a Dryad digital repository: DOI: 10.7272/Q6SJ1HVH. Raw TCR sequencing reads available through NCBI Short Read Archive (SRA) BioProject #PRJNA1110271 with associated BioSample Accession numbers SAMN41334731, SAMN41334732, SAMN41334730, SAMN41334729.

Research involving human participants, their data, or biological material

Policy information about studies with human participants or human data. See also policy information about sex, gender (identity/presentation), and sexual orientation and race, ethnicity and racism.

Reporting on sex and gender	All sex data refers to sex and not gender and is presented in Extended Data Table 1 and Extended Data Table 3.
Reporting on race, ethnicity, or other socially relevant groupings	Race and ethnicity were self-reported and presented in Extended Data Table 1 and Extended Data Table 3.
Population characteristics	Additional characteristics including age and underlying medical conditions are provided in Extended Data Table 1. Detailed clinical data describing the population during the course of illness is presented in Extended Data Table 2.
Recruitment	Patients were recruited through the prospectively enrolling multicenter Overcoming COVID-19 and Taking on COVID-19 Together study in the United States. A total of 292 patients were enrolled into 1 of the following independent cohorts between June 1, 2020 and September 9, 2021: 223 patients hospitalized with MIS-C (199 in the primary discovery cohort, 24 in a separate subsequent validation cohort), 29 patients hospitalized for COVID-19 in either an intensive care or step-down unit (referred to as severe acute COVID-19 in this study), and 45 outpatients (referred to as “at-risk controls” in this study) post-SARS-CoV-2 infections associated with mild or no symptoms. For use as controls in the SARS-CoV-2 specific PhIP-Seq, plasma from 48 healthy, pre-COVID-19 controls were obtained as deidentified samples from the New York Blood Center. These samples were part of retention tubes collected at the time of blood donations from volunteer donors who provided informed consent for their samples to be used for research. We are not aware of any self-selection bias which would alter the results of this study as any patient meeting eligibility criteria at any site was eligible.
Ethics oversight	The study was approved by the central Boston Children’s Hospital Institutional Review Board (IRB) and reviewed by IRBs of participating sites with CDC IRB reliance.

Note that full information on the approval of the study protocol must also be provided in the manuscript.

Field-specific reporting

Please select the one below that is the best fit for your research. If you are not sure, read the appropriate sections before making your selection.

☒ Life sciences ☐ Behavioural & social sciences ☐ Ecological, evolutionary & environmental sciences

For a reference copy of the document with all sections, see nature.com/documents/nr-reporting-summary-flat.pdf

Life sciences study design

All studies must disclose on these points even when the disclosure is negative.

Sample size	Sample size was determined based on sample availability, with the goal of including as many samples as possible. This ultimately led to 199 MIS-C patient samples and 45 at-risk control patient samples. Each sample was included in the initial human proteome-wide PhIP-Seq screen. As many samples as possible were included in subsequent experiments, with some attrition as samples from certain individuals were exhausted. The total number of samples used in each experiment is mentioned in the text and figures. For experiments utilizing patient PBMCs, as many samples were utilized as available in our cohort. This ultimately led to experiments conducted on PBMCs from 11 patients with MIS-C and 10 at-risk controls. Given limited number of PBMCs, the activation induced marker assay for identifying SNX8 autoreactive T-cells was prioritized and performed on all patients. Three patients and three controls had a sufficient quantity of PBMCs and correct HLA-type to perform initial tetramer assays, and three additional MIS-C patients were identified in our biobank for use in isolation of the cross-reactive T cell receptors.
Data exclusions	The activation induced marker assay for detecting SNX8 autoreactive T-cells was run on 11 patients with MIS-C and 10 controls. Data is only provided for 9 of the 11 MIS-C patients, because 2 of the MIS-C samples had insufficient total flow cytometry events to analyze (total of 5,099

	and 4,919 events), and is discussed in the methods.
Replication	Because all experiments were performed on human samples with limited supplies, we were not able to repeat the same experiment in the same individual except for the essential experiment of confirming SNX8 autoreactivity in patients and controls to the peptide containing the identified epitope. We included as many samples as possible in each experiment such that the cases and controls served as "biologically similar" samples to one another. We also performed extensive orthogonal validation experiments to reproduce key findings with additional assays. Given the limited number of patient PBMCs, repeating AIM and tetramer binding assays, and T cell receptor isolation experiments, was not possible.
Randomization	Samples were allocated based on clinical disease category.
Blinding	PhIP-Seq was performed with the experimentalists blinded to the samples. Targeted orthogonal validation experiments were not performed blinded to samples, though they were conducted in relatively high throughput with the majority of experiments utilizing 96-well plates with disease categories intermixed making it unlikely the experimenter could be aware of which sample corresponded to which disease category. PhIP-Seq data was analyzed using unbiased, unsupervised methods, but disease category for each sample was known. Targeted immunoprecipitation experiments were analyzed identically in all samples regardless of category. For experiments with patient PBMCs, the experimenter was not blinded to disease state but analysis was performed blinded to patient disease category.

Reporting for specific materials, systems and methods

We require information from authors about some types of materials, experimental systems and methods used in many studies. Here, indicate whether each material, system or method listed is relevant to your study. If you are not sure if a list item applies to your research, read the appropriate section before selecting a response.

Materials & experimental systems

n/a	Involved in the study
☐	☒ Antibodies
☒	☐ Eukaryotic cell lines
☒	☐ Palaeontology and archaeology
☒	☐ Animals and other organisms
☒	☐ Clinical data
☒	☐ Dual use research of concern
☒	☐ Plants

Methods

n/a	Involved in the study
☒	☐ ChIP-seq
☐	☒ Flow cytometry
☒	☐ MRI-based neuroimaging

Antibodies

Antibodies used	Anti-HiBit positive control antibody (Promega, Madison, WI; #CS2006A01 1:10 dilutions), anti-Myc positive control antibody (Cell Signaling Technology, #2272 S 1:10 dilution) were used. FITC anti-human HLA-A2 Antibody (BioLegend #343303, Clone BB7.2; 1:100 dilution), Alexa 647 conjugated anti-CD3 (BioLegend #317312, Clone OKT3; 1:100 dilution), Alexa 488 conjugated anti-CD4 (BioLegend #317420, Clone OKT4; 1:100 dilution), Alexa 700 conjugated anti-CD8 (BioLegend #344724, Clone SK1; 1:100 dilution), PE-Dazzle 594 conjugated anti-OX-40 (BioLegend #350020, Clone ACT35; 1:100 dilution), PE conjugated anti-CD69 (BioLegend # 310906, Clone FN-50; 1:100 dilution), BV421 conjugated anti-CD137 (BioLegend #309820, 4B4-1; 1:100 dilution), PerCP-Cy5.5 conjugated anti- CD14 (BioLegend #325622, Clone HCD14; 1:100 dilution), PerCP-Cy5.5 conjugated anti- CD16 (BioLegend #360712, Clone B73.1; 1:100 dilution), PerCP-Cy5.5 conjugated anti- CD19 (BioLegend #302230, Clone HIB19; 1:100 dilution), eFluor 506 conjugated Live/dead dye (Invitrogen #65-0866-14, 1:100 dilution), PerCP-Cy5.5 conjugated anti-CD4 (BioLegend #300530, Clone RPA-T4; 1:100 dilution). FITC-conjugated anti-human CD3 (Biolegend #317306, clone OKT3, lot# B390808; 1:20 dilution), BV605-conjugated anti-human CD8 (Biolegend #344742, clone SK1, lot# B371925; 1:20 dilution), BV510-conjugated anti-human CD4 (Biolegend #317444, clone OKT4, lot# B375526; 1:20 dilution), BV510-conjugated anti-human CD14 (Biolegend #367124, clone 63D3, lot# B390770; 1:20 dilution), BV510-conjugated anti-human CD16 (Biolegend #302048, clone 3G8, lot# B372132; 1:20 dilution), BV510-conjugated anti-human CD19 (Biolegend #302242, clone HIB19, lot# B390665; 1:20 dilution), and Ghost Dye Violet 510 Viability Dye (Tonbo Biosciences #13-0870-T500, lot# D0870061322133; 1:400 dilution). PE-conjugated anti-human β2m antibody (Santa Cruz Biotech #sc-13565, clone BBM.1) at 1:200.
Validation	All antibodies were purchased from commercial suppliers including Promega, Cell Signaling Technology, BD, BioLegend, Tonbo, ThermoFisher, Sigma, and eBiosciences with validation data and applicable citations available on product listings for all antibodies (see individual catalog numbers). Antibodies that have previously been validated in the literature were preferred and used at specified dilutions or according to the manufacturer's specifications.

Flow Cytometry

Plots

Confirm that:

☒ The axis labels state the marker and fluorochrome used (e.g. CD4-FITC).

☒ The axis scales are clearly visible. Include numbers along axes only for bottom left plot of group (a 'group' is an analysis of identical markers).

☒ All plots are contour plots with outliers or pseudocolor plots.

☒ A numerical value for number of cells or percentage (with statistics) is provided.

Methodology

Sample preparation

For AIM assay:
Peripheral blood mononuclear cells (PBMCs) were obtained from 10 patients with MIS-C and 10 controls for use in the AIM assay. PBMCs were thawed, washed, resuspended in serum-free RPMI medium, and plated at a concentration of 1e106 cell/well in a 96-well round-bottom plate. For each individual, PBMCs were stimulated for 24-hours with either the SNX8 pool (see above) at a final concentration of 1 ug/mL/peptide in 0.2% DMSO, or a vehicle control containing 0.2% DMSO only. For 4 of the controls and 2 of the MIS-C patients, there were sufficient PBMCs for an additional stimulation condition using the SNX8 high resolution epitope pool (see above) also at a concentration of 1 ug/mL/peptide in 0.2% DMSO for 24-hours. Following the stimulation, cells were washed with FACS buffer (Dulbecco's PBS without calcium or magnesium, 0.1% sodium azide, 2 mM EDTA, 1% FBS) and stained with the following antibody panel for 20 minutes at 4 degrees and then flow cytometry analysis was immediately performed.

For tetramer assay:
PBMCs from 2 MIS-C patients with HLA-A*02.01 (both PAXGene genotyped, 1 confirmed by serotyping) and 1 MIS-C patient with HLA-B*35.01 (PAXGene genotyped), and 3 at-risk controls with HLA-A*02.01 (all 3 identified by serotyping, 2 of 3 confirmed by PAXGene genotyping, other sample did not have gDNA available for genotyping) were thawed, washed, and put into culture with media containing recombinant human IL-2 at 10 ng/mL in 96-well plates. Peptide fragments LQLPQGITL and MQMPQGNPL were then added to PBMCs to a final concentration of 10 ug/mL/peptide and incubated (37C, 5% CO_2) for 7 days.

Following the 7 days of incubation, a total of 8 pMHCI tetramers were generated from UV-photolabile biotinylated monomers, 4 each from HLA-A*02:01 and HLA-B*35:01 (NIH Tetramer Core). Peptides were loaded via UV peptide exchange. Tetramerization was carried out using streptavidin conjugated to fluorophores PE and APC or BV421 followed by quenching with 500uM D-biotin. Tetramers were then pooled together as shown below. All PBMCs were then treated with 100 nM Dasatinib (StemCell) for 30 min at 37 °C followed by staining (no wash step) with the respective tetramer pool corresponding to their HLA restriction (final concentration, 2 to 3 µg/ml) for 30 min at room temperature. Cells were then stained with the cell surface markers for 20 minutes, followed by immediate analysis on a flow cytometer.

For single-cell index sorting of ex vivo and peptide-specific expansion: Unexpanded PBMCs (direct ex vivo) or peptide-expanded T cells were washed in 1x PBS, and treated with 100 nM dasatinib (Sigma-Aldrich #CDS023389) in 1x PBS for 30 min at 37°C and 5% CO_2. Cells were then pelleted and resuspended in 50 µL FACS buffer (1x PBS, 0.04% BSA) supplemented with human TruStain FcX blocking buffer (Biolegend #422302; 1:10 dilution), 500 µM D-biotin (ThermoFisher Scientific #B20656), and a unique tetramer cocktail containing MADS-Tetramer-PE (1:10 dilution), MADS-Tetramer-APC (1:10 dilution), SNX8-Tetramer-PE (1:10 dilution) and SNX8-Tetramer-BV421 (1:10 dilution) based on participant HLA type (A*02:01; A*02:06). Cells were incubated in the dark at 25°C for 1 h followed by direct addition of 50 µL (100 µL total volume) of FACS supplemented with 500 µM D-biotin and an antibody cocktail containing FITC-conjugated anti-human CD3 (Biolegend #317306, clone OKT3, lot# B390808; 1:20 dilution), BV605-conjugated anti-human CD8 (Biolegend #344742, clone SK1, lot# B371925; 1:20 dilution), BV510-conjugated anti-human CD4 (Biolegend #317444, clone OKT4, lot# B375526; 1:20 dilution), BV510-conjugated anti-human CD14 (Biolegend #367124, clone 63D3, lot# B390770; 1:20 dilution), BV510-conjugated anti-human CD16 (Biolegend #302048, clone 3G8, lot# B372132; 1:20 dilution), BV510-conjugated anti-human CD19 (Biolegend #302242, clone HIB19, lot# B390665; 1:20 dilution), and Ghost Dye Violet 510 Viability Dye (Tonbo Biosciences #13-0870-T500, lot# D0870061322133; 1:400 dilution) for 30 minutes in the dark at 4°C. Cells were then pelleted, washed twice with 4 mL FACS buffer (containing 500 µM D-biotin), suspended in 500 µL FACS (containing 500 µM D-biotin), and passed through a 45 µM filter before proceeding to single-cell sorting on a Sony SY3200 cell sorter.

For specificity validation of Jurkat-TCR cell lines: 1x10^6 Jurkat-TCR+ cell lines or untransduced Jurkat J76.7 (TCR-null) were washed in 1x PBS and resuspended in 50 µL FACS buffer (1x PBS, 0.04% BSA) and a unique tetramer cocktail containing MADS-Tetramer-PE (1:10 dilution), MADS-Tetramer-APC (1:10 dilution), SNX8-Tetramer-PE (1:10 dilution), and SNX8-Tetramer-BV421 (1:10 dilution) based on the restricting HLA type (A*02:01; A*02:06). Tetramers conjugated to the Wuhan peptide sequence (LQLPQGTTL), including Wuhan-Tetramer-PE (1:10 dilution) and Wuhan-Tetramer-APC (1:10 dilution), were also tested. A second set of wells were set up in which each individual tetramer was used to stain cells. Cells were incubated in the dark at 25°C for 30 min after which 50 uL of FACS buffer containing Ghost Dye Violet 510 Viability Dye (Tonbo Biosciences #13-0870-T500, lot# D0870061322133; 1:400 dilution) was added for an additional 30 min incubation in the dark at 25°C. Cells were then washed twice with 1 mL FACS buffer and suspended in 300 µL FACS.

For HLA monomer fold testing: Unfolded, biotinylated easYmer monomers (Immudex) were obtained for HLA-A*02:01 and HLA-A*02:06. SARS-CoV-2 MADS (LQLPQGITL), SARS-CoV-2 Wuhan (LQLPQGTTL), and human SNX8 (MQMPQGNPL) peptides were commercially synthesized (Genscript), diluted to 1 mM in ddH2O or DMSO, and loaded onto each easYmer allele according to the manufacturer's instructions at 18°C for 48 h. Proper peptide-HLA monomer formation and MADS and SNX8 peptide binding strength was evaluated for each HLA using a 'β2m fold test' relative to negative (no peptide; unloaded

monomer) and positive (strong binding peptide; CMV pp65 495-503 [NLVPMVATV]) controls as per the manufacturer's protocol. Briefly, peptide-loaded monomers with a concentration of 500 nM were serially diluted to 9 nM, 3 nM, and 1 nM in dilution buffer (1x PBS with 5% glycerol [Sigma-Aldrich #G5516]) and incubated with streptavidin beads (Spherotech #SVP-60-5, 6-8 μm) at 37°C for 1 hour to allow binding of stable complexes to beads, then washed three times with FACS buffer (1x PBS, 0.5% BSA [Sigma-Aldrich #A7030], 2 mM EDTA [ThermoFisher Scientific #15575-038]). Samples were then stained with PE-conjugated anti-human β2m antibody (Santa Cruz Biotech #sc-13565, clone BBM.1) at 1:200 for 30 min at 4° C, washed 3 times with FACS buffer, and analyzed on a 5 Laser 16UV-16V-14B-10YG-8R AURORA spectral cytometer (Cytek).

Instrument

BD LSR Fortessa; Sony SY3200 cell sorter; 5 Laser 16UV-16V-14B-10YG-8R AURORA spectral cytometer (Cytek)

Software

FlowJo v10.7.2 or v10.8.2 was used for the analysis.

Cell population abundance

The final cell population abundance is outlined in Figure 4 and Extended Data Figure 6. Cell population frequency (%parent gate) is detailed in Figure 5b and Extended Data Fig. 10a-c

Gating strategy

For AIM assay:
An initial generous gate was drawn which captured lymphocytes using FSC-A/SSC-A as shown in Extended Data Figure 1A. Singlets were then identified with a FSC-H/FSC-W gate followed by a SSC-H/SSC-W gate. Live cells which were negative for the CD14/CD16/CD19 dump were then gated. T cells were then identified by CD3 surface staining with a clear discrete CD3+ population. CD4 and CD8 cells were then gated on with clear discrete populations. Activated CD4 T-cells were defined as those which were co-positive for OX40 and CD137. Activated CD8 T-cells were defined as those which were co-positive for CD69 and CD137. Gating thresholds for activation were defined by the outer limits of signal in the vehicle controls allowing for up to 2 outlier cells.

For tetramer assay:
An initial generous gate was drawn which captured lymphocytes using FSC-A/SSC-A as shown in Extended Data Figure 1B. Singlets were then identified with a FSC-H/FSC-A gate followed by a SSC-H/SSC-A gate. Dead cells were excluding use a live/dead stain, and CD14/CD16/CD19 positive cells were excluded using a dump gate. CD8 positive surface staining then identified a clear distinct positive population on which to perform the tetramer gating. A stringent tetramer gating strategy was used to identify cross-reactive T-cells, whereby CD8+ T-cells were required to be triple-positive for PE, APC, and BV421 labels (i.e. a single CD8 T-cell bound to PE conjugated LQLPQGITL and/or PE conjugated MQMPQGNPL in addition to APC-conjugated LQLPQGITL and BV421 conjugated MQMPQGNPL). To accomplish this first all PE positive cells were gated on based on identification of outliers from the main CD8+ population. Then a co-positive BV421/APC were identified with an arbitrary gate (insufficient PE+ cells to draw a gate based on distinct cell populations) which was consistent across all samples.

For single-cell index sorting: Single cells were sorted from live (BV510-neg) and lineage (CD4-BV510, CD14-BV510, CD16-BV510, CD19-BV510)-negative, CD3-FITC+/CD8-BV605+ T lymphocytes positive for MADS/SNX8-Tetramer (PE) and MADS-Tetramer (APC) and/or SNX8-Tetramer (BV421) as described in Extended Data Fig. 9a.

For evaluating Jurkat-TCRs: Gates include single, live (BV510-neg), transduced (mCherry+) Jurkat lymphocytes triple positive for MADS/SNX8-(PE), MADS-(APC), and SNX8-(BV421) tetramers as described in Extended Data Fig. 9b.

☒ Tick this box to confirm that a figure exemplifying the gating strategy is provided in the Supplementary Information.
